# High-Resolution Genomic Profiling of *Campylobacter jejuni* Across Human, Livestock, and Yellow-Legged Gull Reservoirs in Croatia Reveals Lineage Segregation and Shared Environmental Genotypes

**DOI:** 10.3390/microorganisms14092000

**Published:** 2026-09-09

**Authors:** Sanja Duvnjak, Luka Jurinović, Fani Krstulović, Viktor Mašović, Andrea Humski, Gordan Kompes, Antonela Bagarić, Eva Postružin, Irena Reil, Merica Carev, Domagoj Drenjančević, Silvija Šoprek Strugar, Arjana Tambić Andrašević, Mirna Vranić-Ladavac, Jasmina Kučinar, Louie Thomas Taylor, Biljana Ječmenica

**Affiliations:** 1 Croatian Veterinary Institute, Savska cesta 143, 10000 Zagreb, Croatia; marjanovic@veinst.hr (S.D.); humski@veinst.hr (A.H.); kompes@veinst.hr (G.K.); marijan@veinst.hr (A.B.); postruzin@veinst.hr (E.P.); reil@veinst.hr (I.R.); 2 Poultry Centre, Croatian Veterinary Institute, Vjekoslava Heinzela 55, 10000 Zagreb, Croatia; fkrstulovic@veinst.hr (F.K.); masovic@veinst.hr (V.M.); jecmenica@veinst.hr (B.J.); 3 Teaching Institute for Public Health, Split-Dalmatia County, 21000 Split, Croatia; mcarev3@gmail.com; 4 Faculty of Health Science, University of Split, 21000 Split, Croatia; 5 School of Medicine, University of Split, 21000 Split, Croatia; 6 ESCMID Food-and-Waterborne Study Group, 4051 Basel, Switzerland; ssoprek@bfm.hr; 7 Department of Clinical Microbiology and Hospital Infections, University Hospital Centre Osijek, 31000 Osijek, Croatia; domagoj.drenjancevic@kbco.hr; 8 Department of Medical Microbiology and Infectology, Faculty of Medicine Osijek, Josip Juraj Strossmayer University of Osijek, 31000 Osijek, Croatia; 9 Division of Parasitology, Department of Clinical Microbiology, University Hospital for Infectious Diseases “Dr. Fran Mihaljević”, 10000 Zagreb, Croatia; 10 School of Medicine, Catholic University Zagreb, 10000 Zagreb, Croatia; 11 Department of Clinical Microbiology, University Hospital for Infectious Diseases “Dr. Fran Mihaljević”, 10000 Zagreb, Croatia; arjana.tambic@bfm.hr; 12 School of Dental Medicine, University of Zagreb, 10000 Zagreb, Croatia; 13 Department of Microbiology, Public Health Institute of Istria County, 52100 Pula, Croatia; mirna.ladavac@zzjziz.hr; 14 Department of Serology and Immunology, Public Health Institute of Istria County, 52100 Pula, Croatia; jasmina.kucinar@zzjziz.hr; 15 Institute for Ornithology, Croatian Academy of Science and Arts, 10000 Zagreb, Croatia; ltaylor@hazu.hr

**Keywords:** WGS, cgMLST/wgMLST, virulome, One Health, *Larus michahellis*

## Abstract

*Campylobacter jejuni* remains a leading cause of foodborne gastroenteritis worldwide, yet the epidemiological role of wild birds as reservoirs remains poorly understood. This study investigated the genetic diversity, transmission dynamics, and virulome architecture of 396 *C. jejuni* isolates collected in Croatia (2021–2025) from human, yellow-legged gull, broiler, bovine, and turkey sources. Whole-genome sequencing revealed high genomic diversity (SI>0.88), encompassing 124 sequence types across 25 clonal complexes. While classical MLST clustered 17.01% (n = 25/147, 95% CI: 11.83–23.82%) of gull isolates with human-associated lineages, high-resolution cg/wgMLST refined this overlap, segregating the majority of yellow-legged gull strains into a distinct, host-adapted ST-1275 clade (51.02%, n = 75/147). Furthermore, cg/wgMLST identified shared multi-host genotypes, such as CT6518 (ST-353) and CT3006 (ST-443), across wild gulls, domestic livestock, and clinical human cases. Virulome screening revealed a conserved pathogenic core alongside a lineage-specific periphery; the Guillain-Barré-syndrome-associated *wla*N gene showed strong restriction to the dominant ST-21 generalist lineage, whereas the Type IV Secretion System was exceptionally rare (<1.5%). These findings demonstrate that, while most yellow-legged gull isolates constitute host-adapted lineages, gulls also harbor overlapping genotypes shared with livestock and clinical human isolates (6.80%, 95% CI: 3.69–12.18%), compatible with shared ecological niches and common contamination sources rather than direct bird-to-human transmission. This highlights their role as environmental sentinels and potential bridge reservoirs within One Health transmission interfaces. This study provides an indispensable baseline for integrated One Health biosecurity and surveillance strategies that bring together poultry production, ecosystem management, and public health infrastructure.

## 1. Introduction

*Campylobacter jejuni* is a significant public health concern for acute diarrheal diseases in humans, as it is one of the leading global causes of such diseases, according to the World Health Organization [1]. As of 2005, campylobacteriosis was the most frequently reported foodborne gastrointestinal infection in the European Union, with an increasing rate and a significant economic burden [2,3]. This bacterium is a common inhabitant of the intestinal microbiota of food-producing animals, including cattle, pigs, and poultry, particularly broilers and turkeys [4,5]. The majority of human infections originate from chicken reservoirs (50–80%), while the remaining proportion (20–30%) is associated with bovines and other sources [3,6]. Transmission routes for *Campylobacter* infections include foodborne pathways, such as the consumption of undercooked poultry, unpasteurized milk, and contaminated fresh produce [7,8], as well as direct contact with infected animals [9]. Additionally, environmental exposure, such as contaminated water sources, whether through agricultural runoff or inadequate sanitation during processing, represents another potential route of infection [10].

Recently, other emerging reservoirs, such as wild birds, have been identified as carriers of potential pathogens affecting both humans and animals. However, their roles are less thoroughly understood due to limited research and difficulties in verifying direct transmission [11,12]. Most studies focus on the prevalence of *Campylobacter* spp. in various wild bird species, and on understanding how wild bird biology influences the prevalence of these bacteria. Migratory species with opportunistic feeding behaviors and occurrence in environments shared with humans and livestock are of particular epidemiological relevance [13,14,15,16,17,18,19,20,21,22].

Recent genome analysis using Multilocus Sequence Typing (MLST) has identified a substantial diversity of *C. jejuni* genotypes originating from a wide range of sources. These have enabled the investigation of genetic variability and evolutionary relationships among strains, providing novel insights into their epidemiology and pathophysiology. Such findings are of critical importance for advancing food safety and protecting public health [23,24]. Studies thus far point to a complex population structure comprising highly genetically diverse host-specific strains, within which a smaller subset population of recurrent genotypes persists and spreads asymptomatically through vectors and reservoirs, leading to clinically relevant human infections [25]. Such strains are found in wild birds, which can introduce them to other populations [26]. Wild birds also serve as reservoirs of novel *Campylobacter* species. They may represent a source of genetic variants that introduce new genes into the gene pool of strains associated with humans or domestic animals [27]. However, wild birds are mostly colonized by species-specific, and thus host-adapted, clonal complexes (CCs), with only a small subset being shared with other hosts [28].

Given the limited data on *Campylobacter* strains circulating in wild birds in Croatia, most research has focused on yellow-legged gulls (*Larus michahellis*), showing a *Campylobacter* occurrence of 14.19%, with *C. jejuni* being the most frequently isolated bacterium [29]. Due to the widespread presence of the yellow-legged gull in Croatia and its adaptation to urban areas, where it lives in proximity to people and feeds opportunistically in both aquatic and terrestrial habitats, enabling pathogen transmission in both directions, this species has the potential to transmit *Campylobacter*. In Croatia, the most frequently isolated genotypes in yellow-legged gulls are host-specific to yellow-legged gulls and belong to CC ST-1275. This complex is well recognized for its association with wild birds, particularly members of the family Laridae. It has been identified in gull populations worldwide, including Australia, the United States, Canada, and Europe, despite the apparent absence of direct physical contact between these populations [29,30,31,32,33]. However, host-generalist genotypes belonging to CCs ST-21 and ST-45 have also been isolated from yellow-legged gulls; these CCs are commonly found in broilers and humans, indicating circulation of shared genotypes between these hosts [29,33].

Building on existing knowledge, this study aims to determine the genomic diversity, population structure, and virulome characteristics of *C. jejuni* across sympatric human, livestock, and yellow-legged gull reservoirs in Croatia, using high-resolution cgMLST and wgMLST. By identifying shared complex types (CTs) and host-segregated lineages, we evaluate the extent of genetic overlap and assess the role of yellow-legged gulls as wildlife reservoirs and environmental sentinels of clinically relevant genotypes. We will determine the sequence types (STs), CCs, and complex types (CTs) circulating in Croatia among yellow-legged gulls, humans, poultry and bovines. The identified strains will be compared across these hosts to evaluate genetic similarity, identify host-specific and generalist genotypes, and assess the presence of virulence-associated genes.

## 2. Materials and Methods

**Figure 1:**
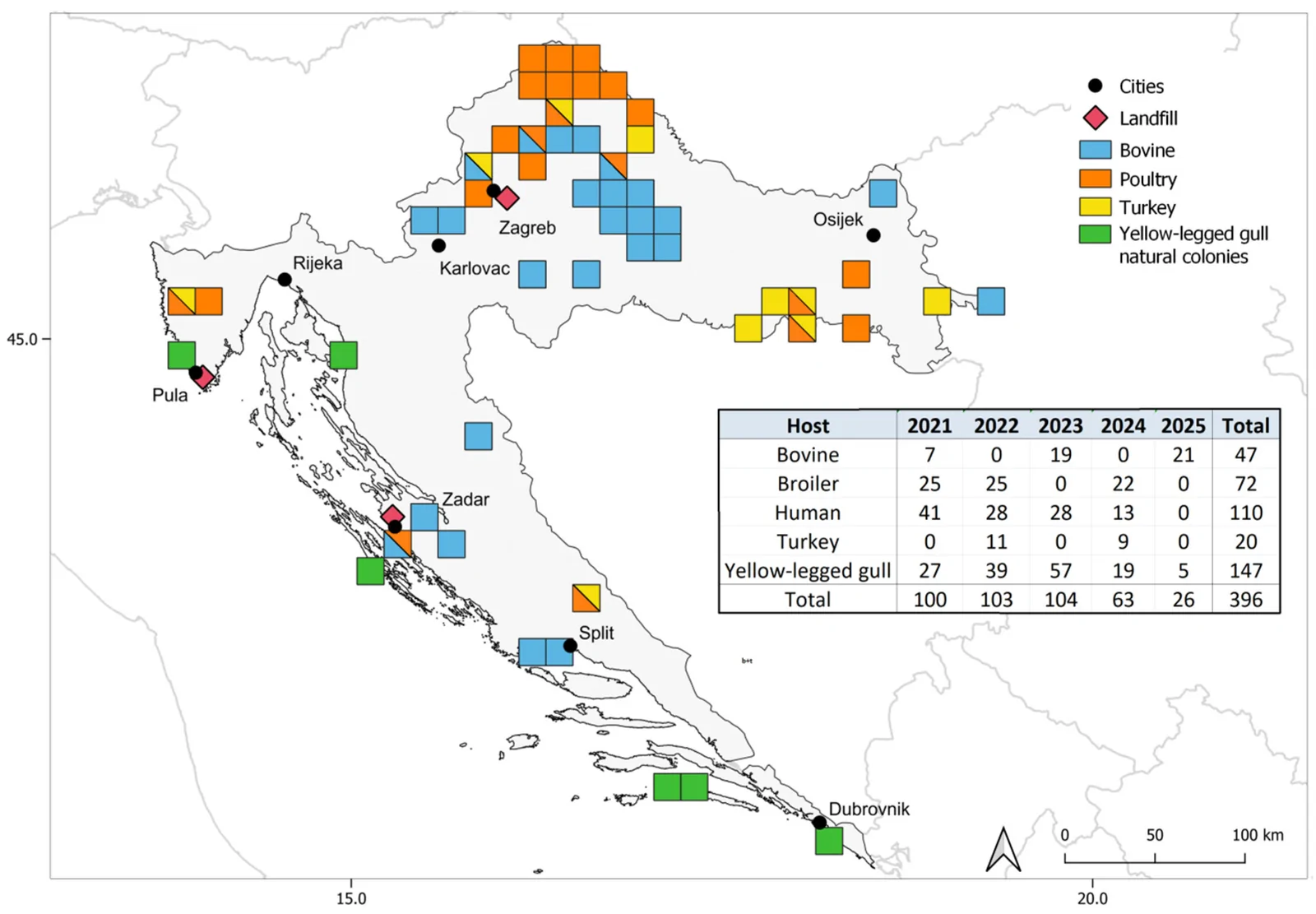
Locations of host sampling with the number of *Campylobacter jejuni* isolates collected from 2021 to 2025. A different color represents each host, while human isolates were collected from four cities (Zagreb, Osijek, Pula and Split).

*Campylobacter jejuni* isolates. Between 2021 and 2025, biological specimens were collected across Croatia from five reservoir groups: yellow-legged gulls, bovines, broilers, turkeys, and humans were collected in different seasons and parts of Croatia (Figure 1). One thousand five hundred and thirty-three (n = 1533) cloacal swabs were collected from yellow-legged gulls from breeding colonies (n = 1320) and municipal landfills (n = 213). To guarantee isolate independence and prevent pseudo-replication, exactly one confirmed *C. jejuni* colony was selected per sampled individual. Clinical human isolates represented non-duplicate, single-patient diagnostic episodes. Broiler, turkey, and bovine isolates originated from distinct flocks or herds under national surveillance programs. Sampling of yellow-legged gulls was conducted across active breeding colonies (n = 109/147, 74.15%) and urban landfill foraging sites (n = 38/147, 25.85%). Detailed specimen metadata, collection years, and host origins are provided in Supplementary Table S1.

Human isolates were collected from gastroenteritis patients with diarrhea symptoms at four independent microbiology laboratories in Croatia, located in Zagreb, Split, Osijek, and Pula (Figure 1). Strains were isolated from primary sterile samples and stool samples.

Isolates from broilers (*Gallus gallus*), turkeys, and bovines were collected during the implementation of national monitoring programs for *Campylobacter*, in accordance with the amendment to Regulation (EC) 2073/2005 introducing a process hygiene criterion for *Campylobacter*, and Regulation (EC) No 2160/2003 [3].

Yellow-legged gulls were captured at breeding colonies (74.15%) and landfills on different sites in the continental and Mediterranean parts of Croatia (25.85%) (Figure 1, Supplementary Table S1). Gulls at landfills were captured using a cannon net. In breeding colonies, adults were captured using ‘walk-in’ traps at active nests with incubating eggs, while nestlings were caught before fledging by hand. Each bird was sampled using cloacal swabs with charcoal (Copan, Italy). Samples were stored at 4 °C and cultured within 3 days to be tested for the presence of thermophilic *Campylobacter* spp., according to the EN ISO 10272-1 method [34].

All strains were revived on blood agar supplemented with 10% defibrinated sheep blood (Columbia agar, Biomerieux, Marcy-l’Étoile, France) and incubated in microaerobic conditions (CampyGen, Thermo Scientific, Waltham, MA, USA) at 42 °C for 24–48 h. A multiplex PCR assay was used to confirm that the strains belonged to *C. jejuni* [35].

A final representative cohort of n = 396 non-duplicate viable isolates (yellow-legged gull: 147; human: 110; broiler: 72; bovine: 47; turkey: 20) was subjected to whole-genome sequencing (WGS). A structured flowchart outlining the process of the WGS analysis is presented in the Supplementary Figure S1.

Genomic DNA extraction was performed by taking a complete loop of fresh culture and dissolving it in 100 µL of Milli-Q water. The mixture was then treated using a NucleoSpin Microbial DNA Mini Kit (Macherey-Nagel, Düren, Germany) according to the manufacturer’s instructions, with the mechanical lysis step at 30 Hz for 12 min on a Tissuelyser (QIAGEN, Hilden, Germany). The quantity and concentration were estimated using a DeNovix DS-11 spectrophotometer (DeNovix, Wilmington, DE, USA) and a Qubit 4.0 fluorometer (Thermo Fisher Scientific, Waltham, MA, USA) with a dsDNA BR assay kit (Life Technologies, Eugene, OR, USA).

Most of the isolates analyzed in this genomic study were newly sequenced for comprehensive whole-genome typing. The exceptions were isolates from 42 human samples that were previously published in Šoprek et al. 2022 [36], and 8 isolates from human, 5 broiler and 2 yellow-legged gull samples because of the potential connection to human isolates published in Šoprek et al. 2023 [23]. The aforementioned isolates were added to this study not to duplicate previously published data, but to expand upon them by providing high-resolution 1602-locus cg/wgMLST and virulome data across the 2021–2025 cohorts.

Genomic Analysis. The extracted DNA was prepared according to the instructions and sent to MicrobesNG (Birmingham, UK) for sequencing. The sequencing was run on an Illumina platform with a 250 bp paired-end output. The results were obtained as raw trimmed reads and assembled fasta files.

The fundamental bioinformatic analysis was provided by MicrobesNG (Birmingham, UK). All obtained reads were put through a standard analysis pipeline. The quality of raw reads was assessed using the FastQC v.0.12.1 tool [37], and the adapter trimming was performed using the Trimmomatic v.0.36 tool [37]. The closest available reference genome was identified using Kraken2 version 2.1.2 (database 01/2026) [38], and the reads were mapped to this using BWA-MEM (Burrows–Wheeler Aligner) [39] to assess the quality of the data. Reads were de novo assembled into contigs using the SPAdes v.3.15.4 assembler [40], and the reads were mapped back to the resultant contigs, again using BWA-MEM v. 0.7.15 to obtain more quality metrics [39]. The quality of the assembled sequences was assessed using the QUAST v.5.2 tool [41], discarding contigs of less than 300 bp, and the initial assemblies met all QC criteria for 345 isolates.

Interspecies contamination was assessed using Kraken2 taxonomic profiling. For 51 samples showing reads matching non-target species, reads were mapped against the *C. jejuni* reference genome NC_002163 to extract target reads prior to de novo reassembly. Only assemblies achieving ≥ 90% core-genome locus call rates were retained. Sensitivity analyses demonstrated no systematic differences in core genome phylogeny or virulome repertoires between the reference-filtered (n = 51) and unfiltered (n = 345) cohorts (*p* > 0.05) (Supplementary Table S2). Consequently, zero genomes were excluded post-sequencing, yielding the final analyzed dataset of n = 396 genomes.

MLST, cg/wgMLST. MBioSEQ Ridom Typer v.12.0.0 software [42] was used for MLST and cgMLST/wgMLST typing, as well as epidemiological analysis of whole-genome sequence data. MLST and cgMLST/wgMLST raw reads with k-mer alignment were mapped against the MLST scheme based on seven housekeeping genes (PubMLST) [43], 637-locus cgMLST, and 958-locus accessory schemes (wgMLST = 1602 loci) sequences to identify the best-matched allele [43]. The MBioSEQ cgMLST has already been proven to be highly congruent with other typing schemes [25]. Epidemiological comparison of the 396 *C. jejuni* genome sequences was performed using a UPGMA radial tree based on a distance matrix of the core genomes of all isolates, pairwise ignoring missing values.

Virulence gene identification. MBioSEQ Ridom Typer v.12.0.0 software [42] was used to screen the *C. jejuni* isolates for 84 potential genes encoding virulence in the VFDB (updated 02/2026) [44] database with a 98% identity threshold and at least 80% coverage. Orange software version 3.40.0 [45] was used for the heatmap analysis using Euclidean distance clustering of virulence factors present and absent in *C. jejuni* according to virulence genes. Dark red dye indicates 100% presence, while white indicates the total absence of a virulence gene.

Statistical Analysis. Statistical analyses were conducted in R (v4.3.2). Categorical variables and gene presence frequencies across host reservoirs were evaluated using two-tailed Fisher’s exact tests or Pearson’s Chi-square (χ^2^) tests. *p*-values were adjusted for multiple hypothesis testing using the Benjamini–Hochberg False Discovery Rate (FDR) procedure α = 0.05). Bivariate associations between specific clonal lineages and virulence determinants were expressed as Odds Ratios (OR) with 95% confidence intervals (95% CI). Proportions and prevalence estimates are reported with Wilson score 95% confidence intervals. Simpson’s Index of Diversity (1-D) with 95% CI was calculated for MLST (ST, CC) and cg/wgMLST schemes. Species richness and sampling saturation across reservoirs were validated using sample-based rarefaction curves (Supplementary Figure S2).

## 3. Results

Extracted DNA. Three hundred and ninety-six *C. jejuni* strains were cultivated and confirmed to be *C. jejuni*. The concentration of extracted DNA ranged from 125 to 378 ng/µL, with a quality of 1.8 to 2.2 for both A_260_/A_280_ and A_260_/A_230_.

Genomic analysis. Samples were de novo assembled into a median contig count of 45 (IQR: 31–58, range: 9–699) with a median assembly coverage of 54x (IQR: 42.1–68.5x) and a median genome size of 1.8 Mb (IQR: 1.72–1.84 Mb). The median N50 of assemblies was 294.4 kb (IQR: 218.1–342.6 kb, range: 182.5–412.8 kb). Assemblies had a median GC content of 30.3% (IQR: 30.1–30.5%). Fifty-one samples showed evidence of interspecies contamination. Reads were mapped against reference strain NC_002163 to isolate *C. jejuni* reads prior to de novo re-assembly. A minimum of 90% core-gene locus calls was required for inclusion.

The sensitivity analysis comparing the full cohort (n = 396) versus the uncontaminated sub-cohort (n = 345) confirmed no reference-mapping bias in core genome clustering or virulome frequencies (*p* > 0.05, Table S3).

Sequence type and clonal complex distribution. The genome analysis covered a total of 1602 genes: 7 housekeeping genes, 637 core genes, and 958 accessory genes. We identified 124 different STs, of which 9 (7.26%) were novel (Table 1), detected in 11 isolates across three different host species. Among the nine novel STs, three exhibited previously unreported alleles: one ST contained a novel allele on *gly*A, while two STs possessed a new allele at the *pgm* locus. All nine novel STs and three new alleles (*gly*A, *pgm*) have been submitted to PubMLST. These STs were mainly present in one isolate, except for two STs, which were present in two isolates. Most of the new STs were identified in yellow-legged gulls (54.55%), broilers (27.27%), and human isolates (18.18%). Simpson’s discriminatory index for MLST typing in this study was 0.979 (95% CI: 0.974–0.983), indicating a high degree of diversity.

The most represented ST was ST6461 (ST-353), detected in 7.07% (n = 28/396) of isolates, followed by ST50 (ST-21; 5.56%, n = 22/396), ST1223 (ST-1275; 5.05%, n = 20/396), ST1268 (ST-1275; 5.05%, n = 20/396), and ST51 (ST-443; 4.80%, n = 19/396). Within CC ST-1275 (n = 75), ST1223 (n = 20/75, 26.67%) and ST1268 (n = 20/75, 26.67%) were dominant and isolated exclusively from yellow-legged gulls. In CC ST-353, ST6461 predominated (53.85%) and was identified mainly in human isolates (60.71%). Similarly, ST50 was the predominant ST within CC ST-21 (37.93%) and was also primarily associated with human isolates (45.45%). In CC ST-443, ST51 was dominant (90.48%) and was most frequently isolated from humans (68.42%).

Strains tested in this study were grouped into 25 different CCs. Simpson’s discriminatory index for the CC typing in this research was 0.885 (95% CI: 0.867–0.903), indicating a high degree of diversity. Twenty-five known STs (21.74%), comprising 50 strains (12.63%), were not assigned to any CC. Eleven strains were part of nine newly defined STs (7.26%). The most abundant CC was ST-1275 (18.94%), which was specific to yellow-legged gulls. ST-21 and ST-353 followed with 14.65% and 13.13%, respectively, while ST-443 accounted for 5.3% of strains.

**Figure 2:**
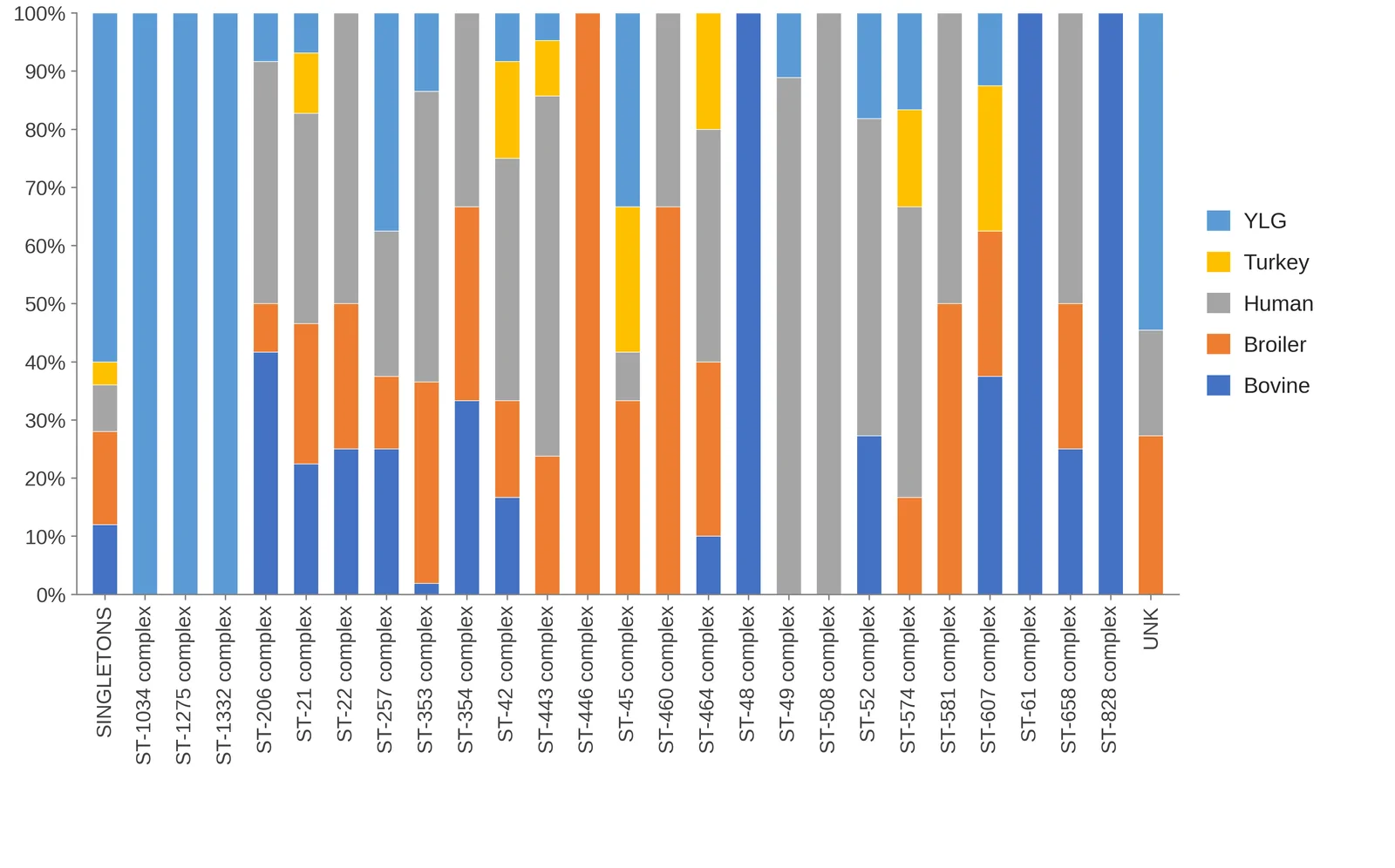
Relative host source distribution (expressed as percentages) of isolates across various multi-locus sequence typing (MLST) clonal complexes (CCs), singletons, and unknown lineages (UNK). The *x*-axis categorizes the isolates by specific CCs, while the *y*-axis indicates the percentage contribution of each source. Colored segments within each stacked bar correspond to distinct host reservoirs. The chart illustrates varying degrees of host specificity, showing lineages predominantly restricted to a single host (such as the ST-1034 complex in YLG or the ST-446 complex in Broiler) alongside highly diverse, multi-host generalist lineages, such as the ST-21 and ST-45 complexes.

The most host-diverse CCs were those identified in all five different hosts tested. ST-21 and ST-42 were found in all hosts. ST-206, ST-257, ST-353, ST-443, ST-45, ST-464, ST-574, and ST-607 in four; and ST-22, ST-354, ST-52, ST-658 in three (Table 1, Figure 2).

ST-21 comprised 36.21% human, 24.14% broiler, 22.41% bovine strains, 10.34% turkey, and 6.90% yellow-legged gull strains, making it a generalist CC. ST-353 comprised 50.00% human and 34.62% broiler strains, while ST-443 comprised 61.90% human strains, making these clonal CCs the ones most frequently associated with human strains (Table 1).

Human isolates showed the highest diversity, comprising 17 different CCs, whereas turkey strains showed the least polymorphism with 7 different CCs (Table 2). Several CCs were represented by single isolates in specific reservoirs, such as ST-508 (n = 1/110) in humans, ST-61 (n = 1/47) and ST-828 (n = 2/47) in bovines, and ST-446 was detected only in broilers (n = 3/72). ST-1034 (n = 8/147, 5.44%), ST-1275 (n = 75/147, 51.02%), and ST-1332 (n = 2/147, 1.36%) were identified exclusively in yellow-legged gull isolates (Table 1).

**Figure 3:**
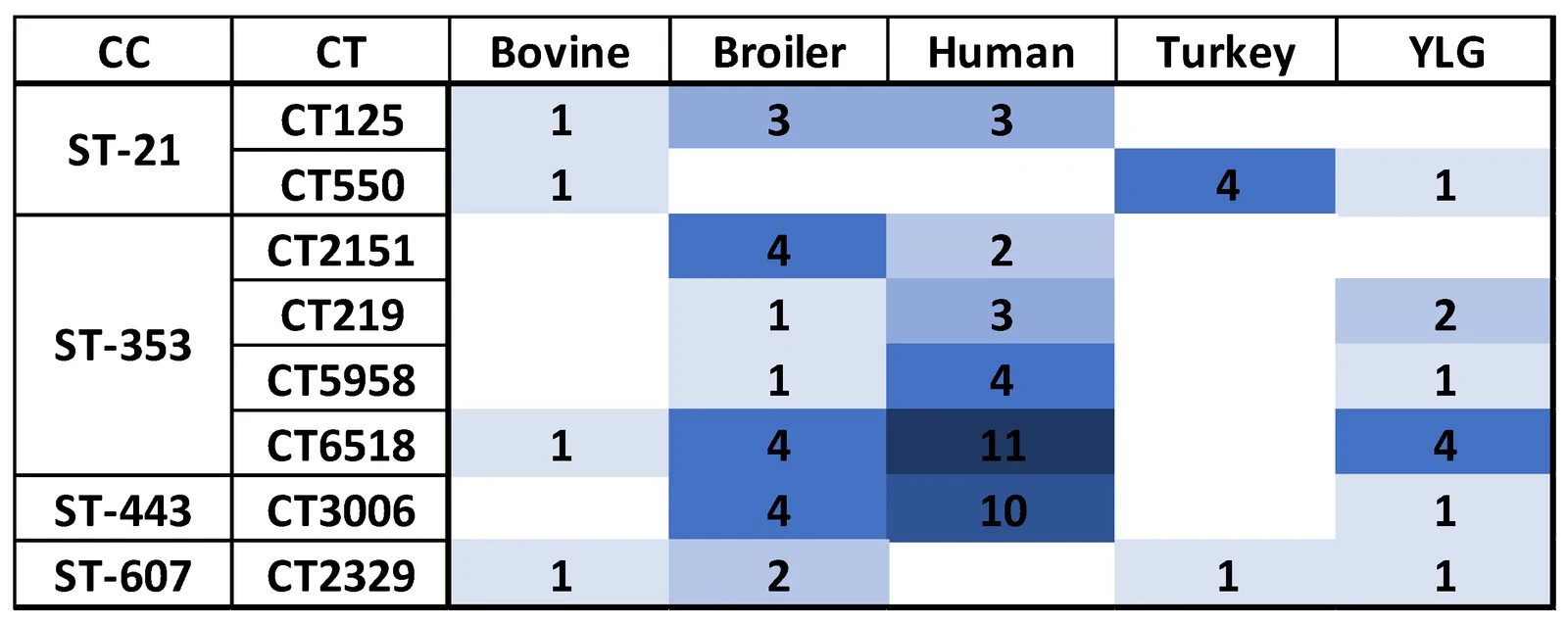
Heatmap representing the distribution and abundance of major complex types (CTs) and corresponding clonal complexes (CCs) across five distinct host reservoirs: human, bovine, broiler, turkey, and gull. Rows depict specific clonal lineages, and columns represent host reservoirs. Cell values and color intensity reflect host-aggregated isolate counts per category (white—0 isolates; light blue—1–2 isolates; deep navy blue—3+ isolates). Shared multi-host genotypes reflect genetic overlap compatible with shared foraging niches or common contamination sources, but they do not imply direct cross-species transmission routes.

**Figure 4:**
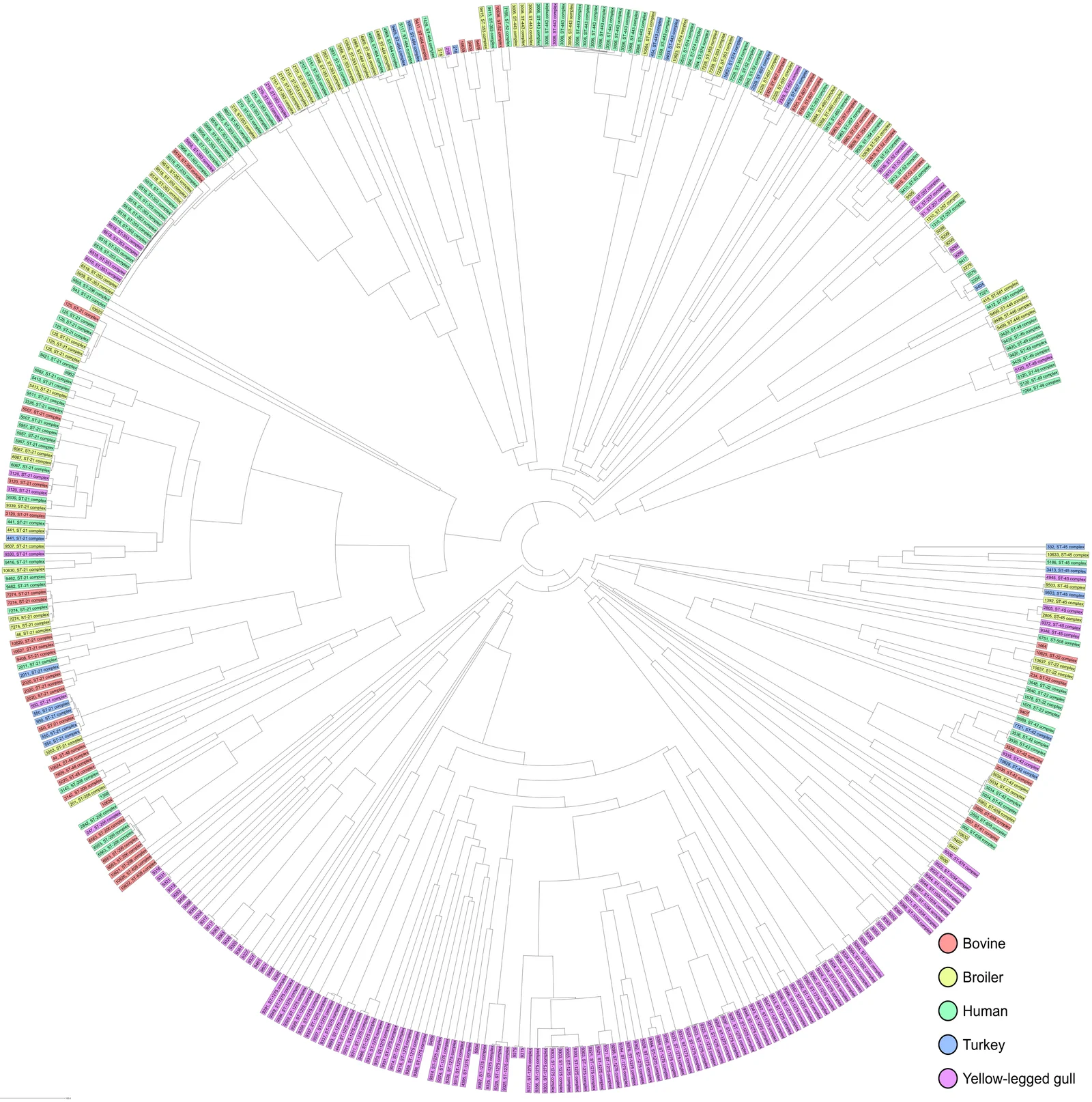
UPGMA radial dendrogram displaying genetic relationships among 396 *Campylobacter jejuni* isolates based on wgMLST analysis across 1602 loci (pairwise missing values ignored). Outer ring labels indicate complex type (CT) and MLST clonal complex (CC). Nodes and branches are color coded according to host source: bovine (red), broiler (yellow), human (green), turkey (blue), and yellow-legged gull (purple). The scale bar represents an allelic distance of 100 core/accessory loci.

cgMLST and wgMLST results. Across the 396 isolates, 219 distinct complex types (CTs) were identified (represented across 222 ST–CT combinations due to three multi-ST complex types). Among these, 178 CTs were distributed across the 25 defined MLST CCs, while 42 CTs belonged to unassigned clonal lineages and novel sequence types (with CT6962 shared between ST-21 and an unassigned clinical isolate). The Simpson’s discriminatory index for cg/wgMLST typing was 0.993 (95% CI: 0.990–0.995). The most prevalent lineages were CT-6518 (ST-353) and CT-3006 (ST-443), representing 5.05% (n = 20) and 3.79% (n = 15) of the total samples, respectively. While several prominent CTs, such as CT-6518, CT-3006, CT-219, CT-550, and CT-2329, exhibited multi-host distribution across human, livestock, and avian reservoirs (Figure 3), the vast majority of the remaining genotypes were restricted to fewer than five isolates spanning only one to three distinct host species. This host-restricted pattern was particularly pronounced among wild bird and yellow-legged gull isolates (Figure 4), which were predominantly identified as host-specific CTs occurring in only one or two samples, with the notable exception of CT-9303 (n = 6) (Supplementary Table S1).

Virulome characterization. A total of 84 virulence genes were identified across the tested *Campylobacter jejuni* isolates (Supplementary Table S3). Twenty-four virulence genes were detected in all tested strains (28.57%), with the majority belonging to functional categories associated with motility (MOT-, n = 45) and chemotaxis (CHEM—*che*A, *che*V, *che*W, *che*Y). Notably, a high prevalence was observed for core genes linked to host cell adhesion (ADH—*cad*F, *jlp*A, *peb*A), cellular invasion (INV—*cia*B, *cia*C, *ept*C), and lipooligosaccharide (LOS) biosynthesis (*gmh*A, *gmh*A2, *gmh*B, *hld*D, *hld*E). Variation was observed in sialylation-associated genes (LOS), including *cst*III (18.69%), capsular polysaccharide (CPS), namely *fcl* (11.36%) and *glf* (10.86%), MOT—flgE2 (6.06%), and the *neu* cluster (*neu*A1—25.51%, *neu*B1 and *neu*C1—26.01%). The Type IV Secretion System (T4SS) gene cluster was least frequently detected in our samples, with a prevalence of 1.26% (n = 5/396, 95% CI: 0.54–2.92%) for *vir*B4, *vir*B8, *vir*B9, and *vir*B10, and 0.51% (n = 2/396, 95% CI: 0.14–1.82%) for *vir*D4.

Broiler isolates harbored a significantly higher overall proportion of target virulence genes (83.55%, n = 5053/6048 locus tests) compared to yellow-legged gull isolates (79.02%, n = 9757/12,348 locus tests; χ^2^ = 54.21, FDR-adjusted *p* < 0.0001).

The molecular-mimicry-associated gene *wla*N was identified in 18.43% (n = 73/396, 95% CI: 14.87–22.59%) of isolates across the total cohort. A strong clonal enrichment was observed within CC ST-21, where 75.86% of ST-21 isolates harbored *wla*N (P (*wla*N^+^|ST-21) = 44/58, 95% CI: 63.49–85.03%), compared to only 8.58% in non-ST-21 lineages (n = 29/338, 95% CI: 6.00–12.11%; Fisher’s Exact Test *p* < 0.0001, OR = 33.49, 95% CI: 16.29–68.82; Table 3). Consequently, ST-21 accounted for 60.27% (P (ST-21|*wla*N^+^) = 44/73, 95% CI: 48.81–70.76%) of all *wla*N-positive isolates detected in this study.

**Figure 5:**
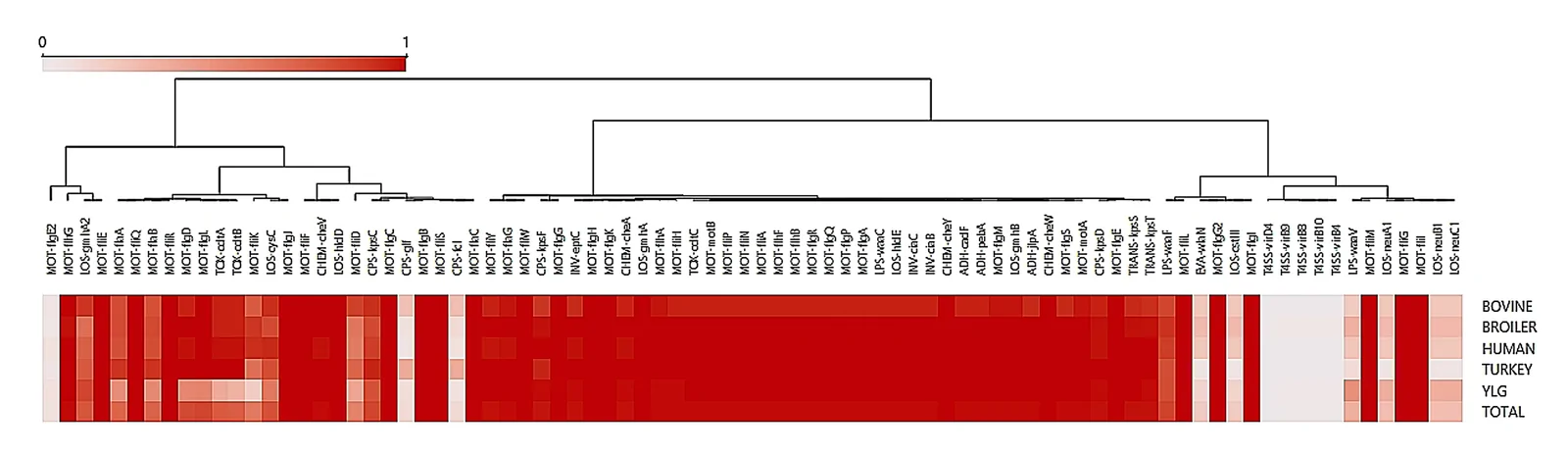
Heatmap and hierarchical clustering (Euclidean distance, average linkage) of host-aggregated virulence gene prevalence across five host reservoirs. Columns represent 84 virulence determinants grouped by functional class: motility (MOT), lipooligosaccharide biosynthesis (LOS), cytolethal distending toxin (TOX), capsular polysaccharide (CPS), chemotaxis (CHEM), invasion (INV), immune evasion (EVA), and Type IV secretion system (T4SS). Rows represent host cohorts and the total study population. Data are presented as host-aggregated prevalence metrics (ranging from 0.0 [0% presence, white] to 1.0 [100% presence, dark red]), rather than single-isolate binary profiles.

The lowest proportion of virulence genes was observed in yellow-legged gull isolates (79.02%), whereas broiler isolates harbored the highest proportion of the screened target genes (83.55%) (Figure 5).

## 4. Discussion

### 4.1. Host Distribution and Genomic Diversity

To understand the population structure and transmission dynamics of *C. jejuni* in Croatia, 396 isolates collected over five years (2021–2025) were analyzed. The dataset encompassed five reservoirs: yellow-legged gulls (37.12%), humans (27.78%), broilers (18.18%), bovines (11.87%), and turkeys (5.05%). Genomic analysis revealed profound diversity, identifying 124 STs, including 9 newly defined STs, grouped into 25 CCs, with 25 STs remaining unassigned. The Simpson’s Diversity Index exceeded 0.8 for both ST and CC designations, consistent with global benchmarks indicating high genomic plasticity within this species [25,46]. Although *C. jejuni* is traditionally considered a fragile, microaerophilic organism, recurrent lineages often demonstrate enhanced survival through biofilm formation and environmental adaptation [25,46]. As *Campylobacter* typically demonstrates host adaptation, cross-species spillover events are relatively infrequent, while being of considerable epidemiological importance [29]. Transmission is heavily mediated by complex environmental networks, including agricultural runoff, wastewater pathways, and insect vectors [23,32]. Wild birds, acting as asymptomatic reservoirs, may contribute to the maintenance and potential dissemination of diverse genotypes across broad ecological niches. However, the presence of matching sequence types across wild birds and domestic animals reflects shared exposure to contaminated environmental sources rather than definitive direct transmission pathways [23].

### 4.2. Lineage Ecology

Core Generalists vs. Host Specialists

The distribution of CCs highlighted distinct patterns of host-specificity and broad generalism across reservoirs; in humans, this was dominated by ST-353 (20.91%), ST-21 (17.27%), and ST-443 (11.82%). ST-508 was uniquely specific to humans. Among poultry hosts, broilers were dominated by ST-353 (25.00%) and ST-21 (19.44%), whereas turkeys predominantly harbored ST-21 (30.00%) and ST-45 (15.00%). In bovines, ST-21 dominated (27.66%), unclassified CCs (12.77%), and ST-206 (10.64%). Yellow-legged gulls harbored host-specific lineages including ST-1034 (5.44%), ST-1275 (51.02%), and ST-1332 (1.36%). Our data showed a high degree of host overlap, particularly within ST-21, a well-known global generalist found across all farm types and seasons [29]. Our bovine and broiler profiles overlapped by 60% with cattle and poultry strains reported in the UK, France, and Korea, where ST-21, ST-61, and ST-48 bridge livestock reservoirs to human infections [29,31,32]. Similarly, the overlap between our broiler and human strains reached 48.61%, revealing a striking 78% concordance with global human clinical genotypes. This mimics trends in Denmark and Switzerland, where poultry-specialist lineages (ST-257, ST-353, ST-354) display prolonged persistence across production cycles due to contaminated processing environments [31,47,48,49].

### 4.3. Resolution of cgMLST and Wildlife Transmission

While seven-gene MLST clustered 17.01% (n = 25/147, 95% CI: 11.83–23.82%) of gull isolates into multi-host clonal complexes shared with humans, high-resolution cg/wgMLST refined this cross-host overlap to 6.80% (n = 10/147, 95% CI: 3.69–12.18%). This marked reduction underscores the superior resolution of whole-genome gene-by-gene typing in resolving false-positive epidemiological linkages generated by the slow evolutionary rate of housekeeping loci. The majority of gull isolates formed a distinct, host-adapted clade dominated by CC ST-1275 (n = 75/147, 51.02%) (Figure 4), mirroring literature suggesting that migratory birds possess lower, host-isolated contamination profiles compared to sedentary birds [31]. Nevertheless, the detection of identical complex types, specifically CT6518 (ST-353) and CT3006 (ST-443), across gulls, poultry, cattle, and diarrheic patients highlights shared exposure niches. Because our cross-sectional genomic data cannot determine the directionality or specific chain of transmission, these shared complex types (e.g., CT6518, CT3006) are compatible with shared ecological niches, common contamination points at anthropogenic foraging grounds (e.g., open landfills, wastewater discharge), or diffuse environmental vectors, rather than direct bird-to-human transmission [46,50,51].

### 4.4. Pathogenic Landscape and Virulome Architecture

The *C. jejuni* virulome exhibited a dual structure: a highly rigid, universally conserved pathogenic core and a highly variable, lineage-specific peripheral virulome.

### 4.5. The Conserved Core Virulome

The machinery required for host colonization was characterized by the near-absolute conservation of determinants governing flagellar motility and chemotaxis. This baseline biological fitness aligns with the collective component paradigm established by Burnham and Hendrixson [52], where these systems drive navigation through nutrient-restricted enteric environments. This core was augmented by a uniform distribution of mucosal interaction factors, matching the near-ubiquitous presence of adhesion (cadF) and translocation (ciaB) mediators observed across human and poultry cohorts in Europe [53,54]. Similarly, foundational backbones for lipooligosaccharide (LOS), lipopolysaccharide (LPS), and capsular polysaccharide (CPS) biosynthesis were heavily conserved. This is consistent with the global characterization of surface polysaccharides as “softcore” or “essential core” elements required for cell wall integrity, environmental survival, and host immune evasion across diverse geographical regions [9,55,56].

### 4.6. Variable Loci, Sequelae, and Plasmid-Mediated Risk

Polymorphic diversity was restricted to specific loci associated with severe clinical sequelae and horizontal gene transfer. The EVA-*wla*N gene, which drives the ganglioside-mimicking structures responsible for Guillain-Barré syndrome, was highly localized, appearing in 75.86% (n = 44/58) of isolates within the clinically dominant ST-21 lineage, with ST-21 comprising 60.27% (n = 44/73) of all *wla*N-positive isolates across the entire collection. This strong clonal restriction represents a significantly higher regional risk compared with the global baseline prevalence of 15.2% noted by Wieczorek et al. [53], echoing localized virulence shifts described by Mohamed et al. [57]. Conversely, mobile virulence determinants were exceptionally rare. Components of the Type IV Secretion System (T4SS), a critical vector for plasmid-mediated virulence and antimicrobial resistance (AMR) amplification, were restricted to just five isolates (<1.5%) primarily associated with yellow-legged gulls and specific broiler lines. This sporadic detection is consistent with global database analyses demonstrating that plasmid-mediated *vir*B/D loci are remarkably rare or absent in many continental populations [55]. While plasmid-borne T4SS gene clusters (*vir*B/*vir*D) were exceptionally rare (<1.5%), this does not imply a low overall risk of antimicrobial resistance dissemination. In *C. jejuni*, primary AMR mechanisms (such as point mutations in *gyr*A and 23S rRNA, or chromosomally integrated transposons) operate independently of T4SS-mediated conjugation. Future WGS resistome profiling and phenotypic susceptibility testing are essential to mapping resistance determinants across these Croatian reservoirs.

### 4.7. Public Health Implications and One Health Interventions

#### Targeted Surveillance and Food Safety

Because the majority of human *Campylobacter* infections in Croatia are attributed to poultry sources, interventions should focus substantially on biosecurity measures within processing plants. Implementing stringent hygiene protocols during slaughter and chilling is vital to curbing cross-contamination from persistent, farm-cycle strains [47]. Concurrently, targeted ecosystem management should address environmental reservoirs. Identical CTs that are shared between wild gulls, agricultural animals, and human patients enable wild bird surveillance at nesting colonies and landfills to provide valuable sentinel data on circulating environmental genotypes, independent of direct transmission routes.

### 4.8. Limitations and Future Outlook

While this study successfully employed high-resolution typing approaches to type *Campylobacter jejuni* strains circulating in Croatia, several limitations should be acknowledged. First, the cross-sectional sampling framework was geographically constrained within Croatia. It featured unequal sample distributions across host species (147 gulls, 110 humans, 72 broilers, 47 bovines, and 20 turkeys), reflecting the opportunistic integration of national surveillance and clinical diagnostics. Second, while high-resolution cgMLST identified shared CTs, genomic clustering without fine-scale spatio-temporal tracking cannot resolve the direction of transmission between wildlife, livestock, and humans. Third, the present study focused on MLST, cg/wgMLST, and virulome profiling; targeted resistome characterization and phenotypic antimicrobial susceptibility testing will be required to define the resistance dynamics in these reservoirs. Although even small sample sizes from less frequently studied hosts can provide valuable insights into critical epidemiological networks [16], future studies should expand both geographic coverage and host diversity. Transitioning toward longitudinal metagenomic surveillance will be essential for monitoring temporal genotype fluctuations, mapping interspecies transmission mechanics, and establishing an integrated, multidisciplinary One Health surveillance framework spanning veterinary, environmental, and public health sectors.

## 5. Conclusions

High-resolution cg/wgMLST demonstrated that *Campylobacter jejuni* populations in Croatia are structured into host-adapted lineages and shared multi-host genotypes. While the majority of yellow-legged gull isolates belong to a host-segregated CC ST-1275 clade (51.02%), a discrete subset (6.80%) shares identical complex types (e.g., CT6518, CT3006) with domestic livestock and clinical gastroenteritis patients. These findings are compatible with shared ecological niches and common contamination sources across anthropogenic interfaces. While our data do not determine the direction of transmission or establish wild birds as direct sources of human infection, gulls serve as informative environmental sentinels. Implementing biosecurity at processing facilities, managing landfill access, and sustaining integrated One Health genomic surveillance remain critical priorities.

## Figures and Tables

**Table 1:** Clonal complexes (CCs) distribution and number of associated sequence types (STs) with isolate counts and host distribution.

CC	No. of Unique STs	Isolates	Host (No. of Isolates)
ST-1034	2	8	yellow-legged gull (8)
ST-1275	15	75	yellow-legged gull (75)
ST-1332	1	2	yellow-legged gull (2)
ST- 206	4	12	bovine (5), broiler (1), human (5), yellow-legged gull (1)
ST-21	12	58	bovine (13), broiler (14), human (21), turkey (6), yellow-legged gull (4)
ST-22	2	8	bovine (2), broiler (2), human (4)
ST-257	4	8	bovine (2), broiler (1), human (2), yellow-legged gull (3)
ST-353	11	52	bovine (1), broiler (18), human (26), yellow-legged gull (7)
ST-354	2	3	bovine (1), broiler (1), human (1)
ST-42	3	12	bovine (2), broiler (2), human (5), turkey (2), yellow-legged gull (1)
ST-443	2	21	broiler (5), human (13), turkey (2), yellow-legged gull (1)
ST-446	1	3	broiler (3)
ST-45	4	12	broiler (4), human (1), turkey (3), yellow-legged gull (4)
ST-460	2	3	broiler (2), human (1)
ST-464	2	10	bovine (1), broiler (3), human (4), turkey (2)
ST-48	4	4	bovine (4)
ST-49	3	9	human (8), yellow-legged gull (1)
ST-508	1	1	human (1)
ST-52	4	11	bovine (3), human (6), yellow-legged gull (2)
ST-574	3	6	broiler (1), human (3), turkey (1), yellow-legged gull (1)
ST-581	1	2	broiler (1), human (1)
ST-607	1	8	bovine (3), broiler (2), turkey (2), yellow-legged gull (1)
ST-61	1	1	bovine (1)
ST-658	3	4	bovine (1), broiler (1), human (2)
ST-828	2	2	bovine (2)
UNDEF	25	50	bovine (6), broiler (8), human (4), turkey (2), yellow-legged gull (30)
	9 NEW	11	broiler (3), human (2), yellow-legged gull (6)

**Table 2:** Host-associated diversity of clonal complexes (CCs) and complex types (CTs) and their sequence types (STs), including STs with unknown CC assignment (novel STs).

Host	No of CCs	No. of STs	Novel STs	No. of CTs	No. of Isolates
Bovine	14	29	0	37	47
Broiler	16	40	3	47	72
Human	17	44	2	66	110
Turkey	7	14	0	17	20
YLG	14	52	6	100	147

**Table 3:** Contingency table showing the distribution of the *wla*N gene in the dataset according to ST-21 CC.

Clonal Complex	*wlaN* Positive	*wlaN* Negative	Total
ST-21 CC	44	14	58
NON-ST-21 CC	29	309	338
Total	73	323	396

Fisher’s Exact Test: *p* < 0.0001; odds ratio (OR) = 33.49 (95% CI: 16.29–68.82).

## Data Availability

The original contributions presented in the study are included in the article/Supplementary Material. Further inquiries can be directed to the corresponding author.

## Supplementary Materials

The following supporting information can be downloaded at: https://www.mdpi.com/article/10.3390/microorganisms14092000/s1, Table S1: Detailed specimen metadata, collection data, host origins and MLST and cgMLST/wgMLST results. Table S2A: Assembly Quality and Core-Genome Locus Call Rates, Table S2B: Virulome Repertoire and Virulence Gene Frequencies, Table S2C: Host and Lineage Distribution Across Cohorts. Table S3. Comprehensive statistical profiling and prevalence of 84 virulence-associated determinants across human, livestock, and yellow-legged gull reservoirs in Croatia (N = 396). Categorical comparisons across the five host reservoirs were evaluated using two-tailed Pearson's Chi-square (χ2) tests or Fisher's exact tests. *p*-values were adjusted for multiple testing across all 84 screened loci using the Benjamini–Hochberg False Discovery Rate (FDR) procedure (*α* = 0.05, q-value). Figure S1. Flowchart of *Campylobacter jejuni* isolate cohort formation and downstream whole-genome sequencing (WGS) genomic profiling. Figure S2. Analytical sample-based rarefaction curves and sampling adequacy assessments for *Campylobacter jejuni* across five host reservoirs in Croatia (2021–2025). (A) Expected Sequence Type (ST) richness [E(S)] plotted against the number of sampled isolates across hosts. Shaded areas represent 95% confidence intervals calculated using analytical variance formulations (Heck et al., 1975). (B) Rarefaction curves for MLST Clonal Complexes (CC), showing complete plateauing and asymptotic saturation of circulating clonal lineages (Good's coverage > 90% in all major hosts. (C) Rarefaction curves for 1,602-locus cg/wgMLST Complex Types (CT), illustrating ongoing fine-scale genomic diversification. (D) Good's sample coverage (%) across host reservoirs for CC, ST, and CT typing resolutions. The red dotted line indicates the 80% coverage threshold for representative epidemiological sampling.
