# Supplementary material for: High-Resolution Genomic Profiling of Campylobacter jejuni Across Human, Livestock, and Yellow-Legged Gull Reservoirs in Croatia Reveals Lineage Segregation and Shared Environmental Genotypes
Source: Microorganisms. 2026 Sep 9;14(9):2000. doi: 10.3390/microorganisms14092000 (PMC13609468; doi:10.3390/microorganisms14092000)
Supplement: Supplementary file 1 [file microorganisms-14-02000-s001.zip › microorganisms-4470725-supplementary .pdf]

Table S2A. Assembly Quality and Core-Genome Locus Call Rates

Statistical comparisons between cohorts using two-tailed Mann-Whitney U test and Welch's unequal variances t-test.

Quality Metric	**Complete Cohort (n=396)**	**Uncontaminated (n=345)**	**Reference-Filtered (n=51)**	***p*-value (n=51 vs. n=345)**	***p*-value (n=396 vs. n=345)**
**Perc. Good Targets (%) (Mean ± SD)**	99.08 ± 0.66	99.13 ± 0.53	98.75 ± 1.19	$p = 0.0140$	$p = 0.5190$
**Median [IQR]**	99.20 [98.70–99.43]	99.20 [98.70–99.50]	99.10 [98.60–99.40]		
**cgMLST Locus Call Rate (%) (Mean ± SD)**	89.93 ± 3.89	89.97 ± 4.00	89.66 ± 3.11	$p = 0.2824$	$p = 0.7778$
**Median [IQR]**	90.03 [87.84–90.91]	90.03 [87.90–90.97]	89.91 [87.68–90.60]		
**Called Core Loci Count (Mean ± SD)**	1434.45 ± 62.10	1435.09 ± 63.78	1430.14 ± 49.56	$p = 0.2824$	$p = 0.7778$
**Median [IQR]**	1436.00 [1401–1450]	1436.00 [1402–1451]	1434.00 [1398–1445]		
**Missing Distance Loci (Mean ± SD)**	160.56 ± 62.10	159.92 ± 63.79	164.86 ± 49.56	$p = 0.2824$	$p = 0.7778$
**Genome Completeness (%) (Mean ± SD)**	99.99 ± 0.01	99.99 ± 0.01	100.00 ± 0.00	$p < 0.0001$	$p = 0.0220$

Table S2B. Virulome Repertoire and Virulence Gene Frequencies

Comparison of key C. jejuni virulence factors across functional categories using two-tailed Fisher's exact test.

Functional Category	Virulence Gene	Locus Tag	Ref-Filtered (n=51)	Uncontaminated (n=345)	Complete Cohort (n=396)	p-value (51 vs. 345)	p-value (396 vs. 345)
**Motility / Flagella**	*flg*B	Cj0527c	50 (98.0%)	345 (100.0%)	395 (99.7%)	*p* = 0.1288	*p* = 1.0000
	*flg*E	Cj0043	51 (100.0%)	345 (100.0%)	396 (100.0%)	*p* = 1.0000	*p* = 1.0000
	*mot*A	Cj1023c	51 (100.0%)	344 (99.7%)	395 (99.7%)	*p* = 1.0000	*p* = 1.0000
**Adherence / Invasion**	*cad*F	Cj1478c	51 (100.0%)	343 (99.4%)	394 (99.5%)	*p* = 1.0000	*p* = 1.0000
	*cia*B	Cj0914c	50 (98.0%)	342 (99.1%)	392 (99.0%)	*p* = 0.4252	*p* = 1.0000
	*peb1*A	Cj0921c	51 (100.0%)	345 (100.0%)	396 (100.0%)	*p* = 1.0000	*p* = 1.0000
	*pld*A	Cj0481	34 (66.7%)	198 (57.4%)	232 (58.6%)	*p* = 0.2264	*p* = 0.7656
**Cytolethal Toxin**	*cdt*A	Cj0498	51 (100.0%)	345 (100.0%)	396 (100.0%)	*p* = 1.0000	*p* = 1.0000
	*cdt*C	Cj0500	43 (84.3%)	289 (83.8%)	332 (83.8%)	*p* = 1.0000	*p* = 1.0000
**Iron Acquisition**	*cfr*A	Cj0755	25 (49.0%)	196 (56.8%)	221 (55.8%)	*p* = 0.3649	*p* = 0.8238
	*chu*A	Cj1614	26 (51.0%)	166 (48.1%)	192 (48.5%)	*p* = 0.7648	*p* = 0.9413
**Iron Acquisition**	*feo*B	Cj1398	46 (90.2%)	315 (91.3%)	361 (91.2%)	*p* = 0.7916	*p* = 1.0000
**Stress Response**	*sod*B	Cj0169	50 (98.0%)	345 (100.0%)	395 (99.7%)	*p* = 0.1288	*p* = 1.0000
	*kat*A	Cj1385	50 (98.0%)	344 (99.7%)	394 (99.5%)	*p* = 0.2413	*p* = 1.0000
	*htr*A	Cj1228c	51 (100.0%)	345 (100.0%)	396 (100.0%)	*p* = 1.0000	*p* = 1.0000
**Chemotaxis**	*che*A	Cj0284c	51 (100.0%)	341 (98.8%)	392 (99.0%)	*p* = 1.0000	*p* = 1.0000
	*che*Y	Cj1118c	51 (100.0%)	345 (100.0%)	396 (100.0%)	*p* = 1.0000	*p* = 1.0000

Global locus-by-locus call rate correlation across all 1,595 loci: Pearson r = 0.9918 ($p < 10^{-15}$).

Table S2C. Host and Lineage Distribution Across Cohorts

χ^2-square test of independence for epidemiological and clonal structure.

Category	Stratification	Ref-Filtered (n=51)	Uncontaminated (n=345)	Total (n=396)	χ2 Test Statistic	p-value
**Host Species**	Bovine	5 (9.8%)	42 (12.2%)	47 (11.9%)	$\chi^2 = 0.8741$	$p = 0.9282$
	Broiler	8 (15.7%)	64 (18.6%)	72 (18.2%)		
	Human	15 (29.4%)	95 (27.5%)	110 (27.8%)		
	Turkey	2 (3.9%)	18 (5.2%)	20 (5.1%)		
	Yellow-legged gull	21 (41.2%)	126 (36.5%)	147 (37.1%)		
**Major Clonal Complexes**	ST-1275 complex	12 (23.5%)	63 (18.3%)	75 (18.9%)	$\chi^2 = 15.3196$ *(for major CCs)*	$p = 0.4287$
	ST-21 complex	7 (13.7%)	51 (14.8%)	58 (14.6%)		
	ST-353 complex	6 (11.8%)	46 (13.3%)	52 (13.1%)		
	ST-443 complex	4 (7.8%)	17 (4.9%)	21 (5.3%)		
	ST-45 complex	3 (5.9%)	9 (2.6%)	12 (3.0%)		
**Major Clonal Complexes**	ST-42 complex	2 (3.9%)	10 (2.9%)	12 (3.0%)		
	ST-206 complex	1 (2.0%)	11 (3.2%)	12 (3.0%)		
	ST-52 complex	2 (3.9%)	9 (2.6%)	11 (2.8%)		
	ST-574 complex	3 (5.9%)	3 (0.9%)	6 (1.5%)		
	UNDEF	9 (17.6%)	52 (15.1%)	61 (15.4%)		

Table S3. Comprehensive statistical profiling and prevalence of 84 virulence-associated determinants across human, livestock, and yellow-legged gull reservoirs in Croatia (N = 396). Categorical comparisons across the five host reservoirs were evaluated using two-tailed Pearson's Chi-square (χ^2) tests or Fisher's exact tests. *p*-values were adjusted for multiple testing across all 84 screened loci using the Benjamini–Hochberg False Discovery Rate (FDR) procedure (α = 0.05, q-value).

Function	Gene	Total (396) % (n) [95% CI]	Bovine (47) % (n)	Broiler (72) % (n)	Human (110) % (n)	Turkey (20) % (n)	Gull (147) % (n)	χ2 (df=4)	Nom. *p*-value	FDR q-value	Reservoir Association / Host Distribution
**Immune Evasion (EVA)**	*wlaN*	**18.43%** (73) [14.87–22.59]	29.79% (14)	22.22% (16)	24.55% (27)	35.00% (7)	6.12% (9)	**25.92**	**3.3 10^{-5}**	**0.0013**	Significant restriction; depleted in wild gulls, enriched in ST-21 hosts
**LOS Biosynthesis & Sialylation**	*cst*III	**18.69%** (74) [15.11–22.87]	12.77% (6)	33.33% (24)	25.45% (28)	20.00% (4)	8.16% (12)	**25.30**	**4.4 10^{-5}**	**0.0013**	Significant enrichment in broiler and human clinical strains
**LOS Sialylation Cluster**	*neu*A1	**25.51%** (101) [21.43–30.07]	21.28% (10)	38.89% (28)	33.64% (37)	25.00% (5)	14.29% (21)	**20.80**	**0.00035**	**0.0039**	Enriched in poultry and human clinical isolates
	*neu*B1	**26.01%** (103) [21.90–30.60]	23.40% (11)	40.28% (29)	33.64% (37)	25.00% (5)	14.29% (21)	**21.62**	**0.00024**	**0.0031**	Enriched in poultry and human clinical isolates
	*neu*C1	**26.01%** (103) [21.90–30.60]	23.40% (11)	40.28% (29)	33.64% (37)	25.00% (5)	14.29% (21)	**21.62**	**0.00024**	**0.0031**	Enriched in poultry and human clinical isolates
**Capsular Polysaccharide (CPS)**	*fcl*	**11.36%** (45) [8.55–14.93]	6.38% (3)	26.39% (19)	13.64% (15)	5.00% (1)	4.76% (7)	**25.02**	**5.0 10^{-5}**	**0.0013**	Significant poultry-associated lineage enrichment

Function	Gene	Total (396) % (n) [95% CI]	Bovine (47) % (n)	Broiler (72) % (n)	Human (110) % (n)	Turkey (20) % (n)	Gull (147) % (n)	χ2 (df=4)	Nom. *p*-value	FDR q-value	Reservoir Association / Host Distribution
**Capsular Polysaccharide (CPS)**	*glf*	**10.86%** (43) [8.11–14.39]	4.26% (2)	25.00% (18)	13.64% (15)	5.00% (1)	4.76% (7)	**24.22**	**$7.2\ 10^{-5}$**	**0.0014**	Significant poultry-associated lineage enrichment
	*kps*C, *kps*D, *kps*F, *kps*S, *kps*T	**100.00%** (396) [99.04–100]	100% (47)	100% (72)	100% (110)	100% (20)	100% (147)	0.00	1.000	1.000	Conserved capsule backbone core
**Motility Loci (MOT)**	*flg*E2	**6.06%** (24) [4.03–8.99]	0.00% (0)	8.33% (6)	10.00% (11)	10.00% (2)	3.40% (5)	8.16	0.086	0.448	Lineage-associated structural variant
	*fla*A, *flaB*, *fla*C, *flg*A-D, *flg*E, *flg*G-K, *flg*P-S, *flh*A-B, *flh*F, *fli*A, *fli*F, *fli*H-N, *fli*P-R, *fli*S, *fli*W, *fli*Y, *mot*A, *mot*B (39)	**100.00%** (396) [99.04–100]	100% (47)	100% (72)	100% (110)	100% (20)	100% (147)	0.00	1.000	1.000	Ubiquitous flagellar basal body & motor core

Function	Gene	Total (396) % (n) [95% CI]	Bovine (47) % (n)	Broiler (72) % (n)	Human (110) % (n)	Turkey (20) % (n)	Gull (147) % (n)	χ2 (df=4)	Nom. *p*-value	FDR q-value	Reservoir Association / Host Distribution
**Capsular Polysaccharide (CPS)**	*flg*G2, *flh*G, *fli*I	**98.74–99.75%** (391–395)	100% (47)	100% (72)	99.09% (109)	100% (20)	98.64% (145)	1.84	0.765	0.950	Highly conserved motility accessory loci
**Chemotaxis (CHEM)**	*che*A, *che*V, *che*W, *che*Y (4)	**100.00%** (396) [99.04–100]	100% (47)	100% (72)	100% (110)	100% (20)	100% (147)	0.00	1.000	1.000	Universal enteric navigation core
**Adhesion (ADH)**	*cad*F, *jlp*A, *peb*A (3)	**100.00%** (396) [99.04–100]	100% (47)	100% (72)	100% (110)	100% (20)	100% (147)	0.00	1.000	1.000	Essential mucosal colonization core
**Invasion (INV)**	*cia*B, *ept*C	**100.00%** (396) [99.04–100]	100% (47)	100% (72)	100% (110)	100% (20)	100% (147)	0.00	1.000	1.000	Universally conserved invasion apparatus
	*cia*C	**98.23%** (389) [96.38–99.16]	97.87% (46)	98.61% (71)	99.09% (109)	100% (20)	97.96% (144)	0.74	0.946	0.985	High conservation across all reservoirs
**Cytolethal Toxin (TOX)**	*cdt*C	**100.00%** (396) [99.04–100]	100% (47)	100% (72)	100% (110)	100% (20)	100% (147)	0.00	1.000	1.000	Universal holotoxin subunit
	*cdt*A, *cdt*B	**97.73%** (387) [95.73–98.81]	97.87% (46)	98.61% (71)	99.09% (109)	100% (20)	95.92% (141)	2.52	0.641	0.950	Highly conserved holotoxin complex
**Core LPS/LOS Backbone**	*gmh*A, *gmh*A2 *gm*B, *hld*D,	**99.75–100.00%** (395–396)	100% (47)	100% (72)	100% (110)	100% (20)	99.32% (146)	1.70	0.791	0.950	Structural cell-wall integrity core

Function	Gene	Total (396) % (n) [95% CI]	Bovine (47) % (n)	Broiler (72) % (n)	Human (110) % (n)	Turkey (20) % (n)	Gull (147) % (n)	χ2 (df=4)	Nom. *p*-value	FDR q-value	Reservoir Association / Host Distribution
	*hld*E, *cys*C, *waa*C, *waa*F (8)										
**Core LPS/LOS Backbone**	*waa*V	**95.45%** (378) [92.90–97.13]	95.74% (45)	97.22% (70)	95.45% (105)	95.00% (19)	94.56% (139)	0.86	0.930	0.985	Conserved LOS transferase
**Type IV Secretion (T4SS)**	*vir*B4, *vir*B8, *vir*B9, *vir*B10	**1.26%** (5) [0.54–2.92]	0.00% (0)	2.78% (2)	0.00% (0)	0.00% (0)	2.04% (3)	3.86	0.425	0.900	Plasmid pVir locus; rare across all hosts
	*vir*D4	**0.51%** (2) [0.14–1.82]	0.00% (0)								

Overall Virulome Functional Burden Across Reservoirs

- **Broiler Cohort (n = 72):** 83.55% overall locus test positivity (n = 5 053 / 6,048 locus tests).
- **Human Cohort (n = 110):** 81.71% overall locus test positivity (n = 7 550 / 9 240 locus tests).
- **Turkey Cohort (n = 20):** 81.43% overall locus test positivity (n = 1 368 / 1 680 locus tests).
- **Bovine Cohort (n = 47):** 80.22% overall locus test positivity (n = 3 167 / 3 948 locus tests).
- **Yellow-Legged Gull Cohort (n = 147):** 79.02% overall locus test positivity (n = 9 757 / 12 348 locus tests).

Statistical Comparison (Broiler vs. Gull): $\chi^2 = 54.21$, df = 1, Benjamini–Hochberg FDR-adjusted $p < 0.0001$.

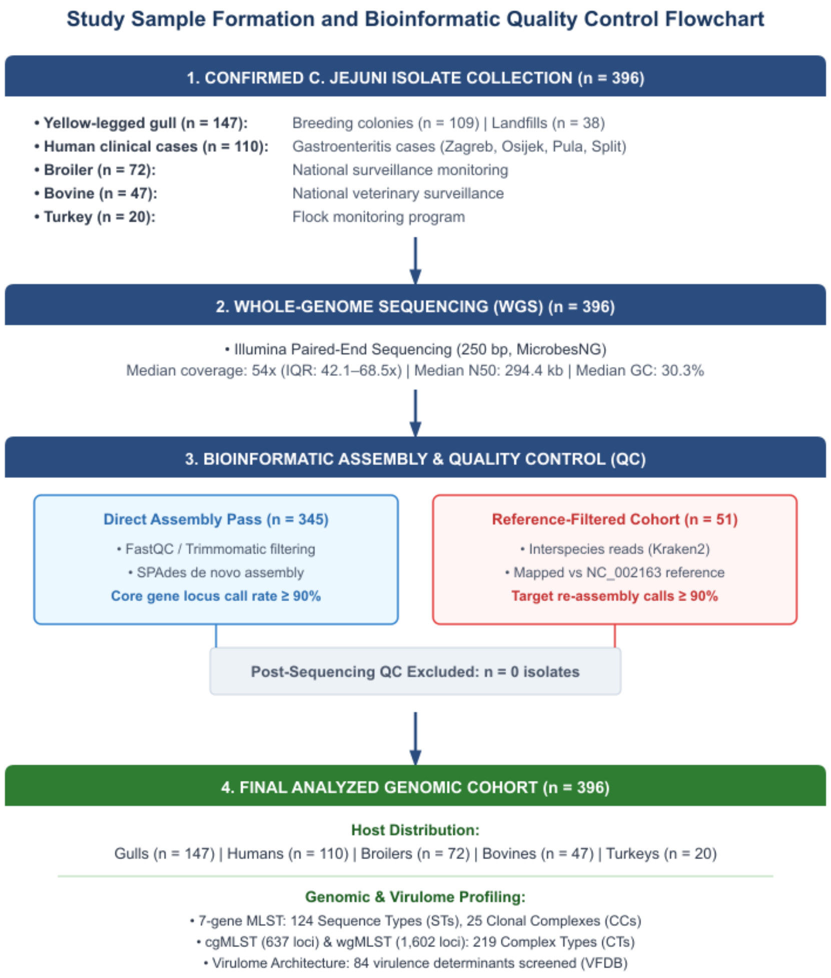

Supplementary Figure S1. Flowchart of Campylobacter jejuni isolate cohort formation and downstream whole-genome sequencing (WGS) genomic profiling

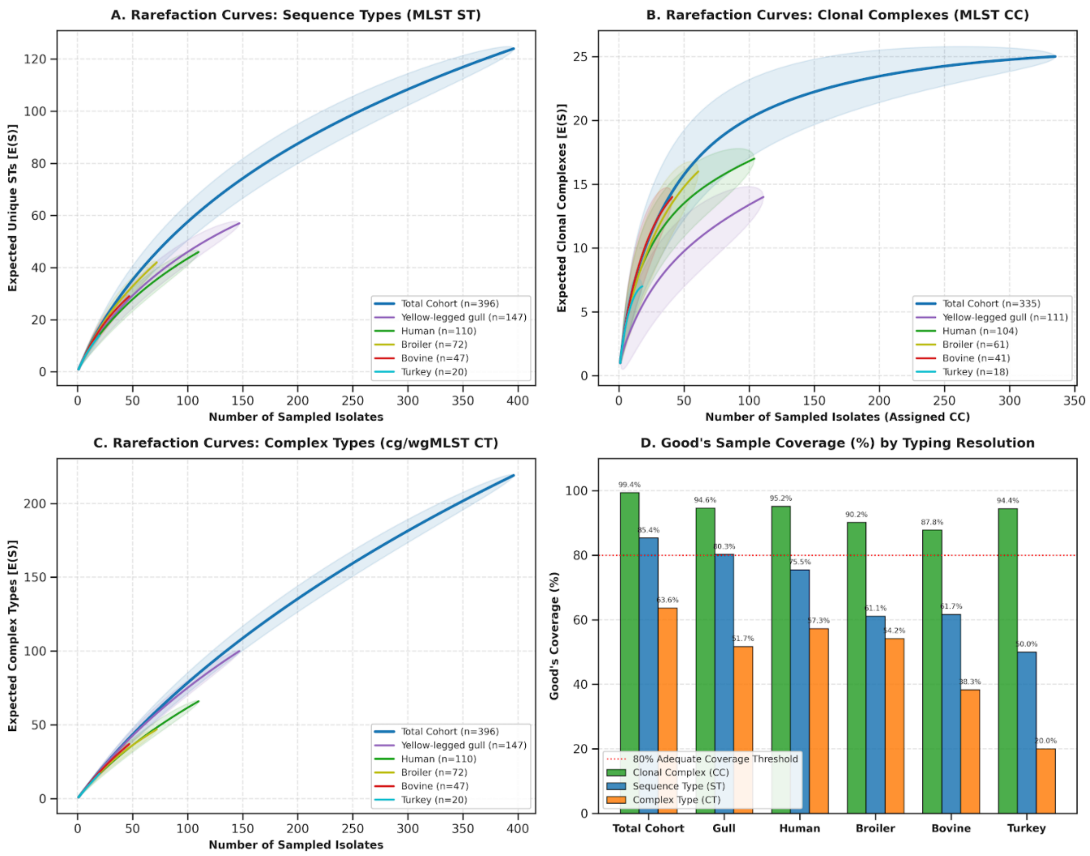

Supplementary Figure S2. Analytical sample-based rarefaction curves and sampling adequacy assessments for Campylobacter jejuni across five host reservoirs in Croatia (2021–2025). (A) Expected Sequence Type (ST) richness [E(S)] plotted against the number of sampled isolates across hosts. Shaded areas represent 95% confidence intervals calculated using analytical variance formulations (Heck et al., 1975). (B) Rarefaction curves for MLST Clonal Complexes (CC), showing complete plateauing and asymptotic saturation of circulating clonal lineages (Good's coverage > 90% in all major hosts. (C) Rarefaction curves for 1,602-locus cg/wgMLST Complex Types (CT), illustrating ongoing fine-scale genomic diversification. (D) Good's sample coverage (%) across host reservoirs for CC, ST, and CT typing resolutions. The red dotted line indicates the 80% coverage threshold for representative epidemiological sampling.
